# Solid-State Hydrogen Storage in Atomic Layer Deposited α‑MoO_3_ Thin Films

**DOI:** 10.1021/acs.energyfuels.5c01159

**Published:** 2025-06-04

**Authors:** David Maria Tobaldi, Salvatore Mirabella, Gianluca Balestra, Daniela Lorenzo, Vittorianna Tasco, Maria Grazia Manera, Adriana Passaseo, Marco Esposito, Andreea Neacsu, Viorel Chihaia, Massimo Cuscunà

**Affiliations:** † CNR Nanotec, Institute of Nanotechnology, University Campus Ecotekne, Via per Monteroni, 73100 Lecce, Italy; ‡ Dipartimento di Fisica e Astronomia ″Ettore Majorana″, Università di Catania, and CNR-IMM, Catania Università, Via Santa Sofia 64, 95123 Catania, Italy; § Department of Mathematics and Physics ‘Ennio De Giorgi”, University of Salento, c/o Campus Ecotekne, Via Monteroni, 73100 Lecce, Italy; ∥ CNR IMM, Institute for Microelectronic and Microsystems, University Campus Ecotekne, Via per Monteroni, 73100 Lecce, Italy; ⊥ Institute of Physical Chemistry “Ilie Murgulescu”, Romanian Academy, Splaiul Independentei 202, 060021 Bucharest, Romania

## Abstract

Hydrogen is an energy vector capable of storing and supplying large amounts of energy, maximizing the benefits of renewable and sustainable energy sources. Hydrogen is usually stored as compressed hydrogen gas or liquid hydrogen. However, the former requires high pressure and the latter cryogenic temperatures, being a huge limit to the widespread adoption of these storage methods. Thus, new materials for solid-state hydrogen storage shall be developed. Here, we show that an α–MoO_3_ thin film, grown via atomic layer deposition, is a material with potential for reversibly storing hydrogen. We found that hydrogen plasma is a convenient way to hydrogenate – at room temperature and relatively low pressures (200 mTorr) – layered α–MoO_3_ thin films. Density functional theory calculations of stepwise hydrogen insertion into α–MoO_3_ reveal that hydrogen atoms preferentially form covalent bonds with monovalent oxygen atoms located within the van der Waals gaps separating the [010]-oriented layers. The hydrogen absorption process has been found to be totally reversible, with desorption of hydrogen effective at 350 °C/4 h under a nitrogen atmosphere, and recoverable after repeated cycles. Furthermore, a nominal 13 nm Al_*x*_O_*y*_ capping layer, grown via atomic layer deposition, has been shown to be efficient in preventing hydrogen release. The volumetric hydrogen storage capacity of 28 kg·m^–3^ achieved in our films is comparable to that of pressurized steel cylinders, highlighting their potential for practical applications. Our essay could be a starting point to a transition from conventional (gas and liquid) to more advantageous solid-state hydrogen storage materials.

## Graphical abstract

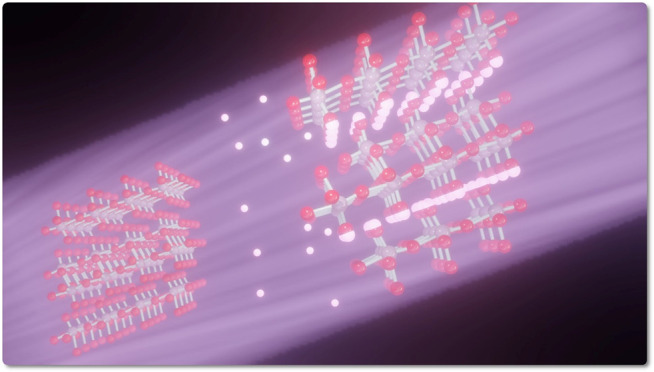

## 1 Introduction

Most of the energy exploited by human-kind has been essentially achieved by using fossil-fuels.^1^ While this boosted the *Industrial Revolution* leading to a global economic growth, the release of greenhouse gases contributed to the environmental concerns we are experiencing nowadays.^2^ A new collective conscience is globally spreading to replace the old carbon-based society with a carbon-neutral one. As a consequence, United Nations’ *Net Zero Coalition* aims to reduce greenhouse gas emissions by 45% by 2030, to reach net zero by 2050.^3^ To this aim, the challenge is providing a sustainable supply of clean and green energy, as the new normal shall follow the regime of renewable energy sources.^4^ Hydrogen, being a zero-emission fuel, is the ideal candidate to replace fossils-fuels. Being green and clean, storable, and transportable, it is foreseen to play an important role in solving energy and environmental issues,^5^ as proven by the growing focus on hydrogen energy research.^6^ Hydrogen-based energy storage systems are gaining momentum as a cost-effective solution for a large-scale renewable energy storage, transport and export.^7^ However, being a fuel itself, it naturally comes together with some degree of safety concerns, as it is conventionally stored at high-pressure as a gas, or as a liquid at cryogenic temperatures.^8^ The primary risk is therefore associated with leakage or boil-off losses, as hydrogen is highly prone to spontaneously combust.^9^

Considering the energy crisis we are facing, storage is one serious bottleneck to overtake. Indeed, safer and alternative materials for solid-state hydrogen storage are strongly desired. Materials-based hydrogen solid-state storage devices are a captivating alternative.^10^ However, most of those based on chemisorption (i.e., hydrides, nitrides, imides) are generally costly, and have irreversible hydrogen absorption/desorption processes.^11^ Additionally, some of these materials involve very high temperatures for hydrogen release.^12^ Thus, advances in energy storage necessitate materials engineered at the nanoscale,^13^ while adhering to principles of sustainability.^14^ Materials having a (2D) layered structure, are drawing attention as candidates to energy storage devices.^15^ Their layered architecture, combined with the multiphase interfaces formed during hydrogen sorption, is considered to enhance hydrogen storage properties.^16^ Among these 2D layered material, molybdenum oxide (MoO_3_) is one of the most appealing oxide,^17^ as also recently proven by Goncalves et al. by means of computational investigation.^18^ While α–MoO_3_ exhibits a lower gravimetric hydrogen storage capacity than materials such as metal hydrides (i.e., 0.7 wt % Vs 7.6 wt % in [MgH_2_][Bibr ref19]), it offers several advantages that make it an attractive alternative. Its layered structure, adaptable hydrogenation behavior, and ability to absorb hydrogen at relatively low pressures provide new possibilities for solid-state hydrogen storage. Moreover, a key challenge in hydrogen storage remains its inherently low volumetric energy density under standard conditions, which is typically addressed through high-pressure storage (*e.g*., 350 or 700 bar).^20^ α–MoO_3_ might represent a suitable oxide-based alternative, as absorption is predicted to be energetically favorable even at ambient temperature and low H_2_ pressures.^18^

MoO_3_ crystallizes in several polymorphs, the orthorhombic molybdite (α–MoO_3_) being the thermodynamically stable and natural occurring of them.^21^ Crystal structure of α–MoO_3_ is described in the space group *Pbnm* (with 4 formula units per unit cell, *Z* = 4), and comprises a bilayer network of distorted edge-sharing MoO_6_ octahedra,^22^ bonded to adjacent layers by van der Waals forces, and stacked along the [010] crystallographic direction.^23^ Small ions are prone to accommodate themselves in the van der Waals gap creating oxygen-deficient α–MoO_3_.^24^ Being hydrogen the smallest of elements, it is totally fit to be inserted into the van der Waals gap.^25^ While MoO_3_ has been broadly adopted as an answer to the electrochemical energy storage dilemma,^26–29^ these hydrogen-ion storage solutions proved themselves to fulfill a limited function in the renewable energy quest.^30^ Solid-state hydrogen storage seems to be a more viable tool, in spite this field being quite new when contrasted with batteries or catalysis studies.^31^ Literature reports about α–MoO_3_ hydrogenation are indeed quite limited. α–MoO_3_ has been hydrogenated as loosen powders,^32^ 2D layered crystals,^33^ or thermally evaporated polycrystalline films.^34^ Hydrogenation is usually assessed by flowing (pure or diluted in N_2_, forming gas) H_2_ gas in heated furnaces,^33,34^ or achieved by plasma reactions in a plasma enhanced chemical vapor deposition system at 180 °C.^32^ While hydrogen plasma has been employed, this has been done to make α–MoO_3–*x*_ nanopowders to be applied as cathode material to lithium ion batteries.^35^

In this work, to fill a literature gap, hydrogen plasma was used at room temperature to hydrogenate α–MoO_3_ thin films oriented along the [010] direction. Although hydrogen plasma treatment may appear energy-intensive, conventional hydrogenation methods generally demand high temperatures and elevated pressures,^36^ both of which contribute significantly to the overall energy consumption of the process. Films were deposited via plasma enhanced atomic layer deposition (PEALD),^37,38^ an extremely versatile technique,^39^ compared to (yet adaptable to functionalize) powdered materials.^40^

Results showed that our films achieved a volumetric hydrogen storage capacity of 28 kg·m^–3^. This is comparable to the capacity of pressurized steel gas cylinders,^19^ which attain a volumetric density of 36 kg·m^–3^ by compressing hydrogen gas to 700 bar.^41^ A nominal 13 nm Al_*x*_O_*y*_ capping layer, grown via atomic layer deposition, has proven effective in preventing hydrogen release. Additionally, the hydrogen sorption process has been found to be totally reversible and recoverable after repeated cycles. To gain deeper insight into the mechanisms governing hydrogen interaction with α–MoO_3_, we conducted density functional theory (DFT) calculations.

## 2 Experimental Section

### 2.1 Film Growth and Hydrogen Plasma Treatment

α–MoO_3_ thin films were deposited over *p*–Si (100) substrates by means of PEALD, following a growing procedure that we developed earlier.^37^ The substrate temperature was set at 400 °C, so as to have [010] oriented α–MoO_3_ films with around 34 nm in thickness. Capping layers of amorphous Al_*x*_O_*y*_, with nominal thickness of 13 and 39 nm, were grown over the α–MoO_3_ via PEALD using trimethylaluminum as the Al source, setting the temperature of the substrate at 100 °C.

Hydrogen plasma treatment was performed, on the Al_*x*_O_*y*_/α–MoO_3_/Si films, in a parallel-plate reactive ion etching reactor (IONVAC, IT). It uses a 13.56 MHz radio frequency signal to generate hydrogen plasma. Specimens were subjected to hydrogen plasma cycles of 12, 24, 48, and 96 min with the pressure in the chamber at 200 mTorr, and keeping the applied power constant at 80 W. Given the large diameter of the parallel plates (20 cm), the applied power density is relatively low, around 0.25 W·cm^–2^.

### 2.2 Characterization

Before, and immediately after being subjected to plasma treatment, α–MoO_3_ films were analyzed by means of X-ray diffraction (XRD) techniques. Grazing incidence XRD (GIXRD) was used to detect the mineralogy of plasma treated films. This was done at room temperature on a Malvern PANalytical X’Pert Pro MRD diffractometer equipped with a fast PiXcel detector using Cu Kα radiation generated at 40 kV and 40 mA. GIXRD patterns were recorded at an incident angle of 0.5°, with a step size of 0.01°2θ, a counting time of 0.5 s, over the 5–30°2θ interval. All of the GIXRD measurements were assessed orienting the films along the (100) reflection of the Si substrate, used as reference peak, to have reliable values of the α–MoO_3_ unit cell parameter *b*. Spectroscopic ellipsometry (SE) was used to retrieve information about the thickness of the films. Measurements were performed on a J.A. Woollam M-2000 ellipsometer at different incident angles (50°, 55°, 60°) over the wavelength range of 450–850 nm. The model used for fitting the SE data comprised four layers (from bottom to top): silicon as the substrate,^42^ native silicon oxide,^43^ MoO_3_ and Al_*x*_O_*y*_ as the top layers. In a simplified picture, Cauchy model, typically used for ALD oxides, was applied to fit the refractive indices of the MoO_3_ and Al_*x*_O_*y*_ layers.^44^ Each fit was considered reliable if the mean square error (MSE), defined as the mean of the squared differences between experimental and estimated values, was below 10. Scanning electron microscopy (SEM) micrographs were acquired using a Zeiss Merlin system operating at an accelerating voltage of 5  kV.

Elastic recoil detection analysis (ERDA) in glancing angle geometry was assessed to determine the amount of hydrogen absorbed in the films. A 2.4 MeV He^+^ beam was directed at the sample surface at an angle of 75° to the normal, and the energy spectrum of forward scattered atoms was measured at an angle of 30° from the incident beam axis. A Mylar foil was placed in front of the detector to stop the scattered He ions, allowing for the acquisition of the energy spectrum of H recoiled atoms. The quantification of H adsorbed in the films was carried out by analyzing the ERDA spectra and using two reference samples with independently measured hydrogen profile.^45^

### 2.3 Computational Details

Molybdite has a layered crystal structure, with four formula units per unit cell, as shown in Figure 1. Within a unit cell, each MoO_6_ octahedron has two oxygen atoms (in position O2) forming asymmetric bonds with two Mo atoms in the *a* direction. Additionally, there are three O3 oxygen atoms in a 3-fold symmetry, creating two short bonds with two Mo atoms in the [001] direction and one longer bond with a Mo atom in the [010] direction.^46^ The sixth oxygen in the MoO_6_ octahedron is the apical oxygen (in position O1) pointing perpendicular to the van der Waals gap (inset of Figure 1).^33^ This configuration enables two van der Waals gaps per α–MoO_3_ unit cell (yellow planes in Figure 1). In each unit cell, a bilayer of two MoO_6_ octahedra is positioned between these two van der Waals gaps.

**Figure 1:**
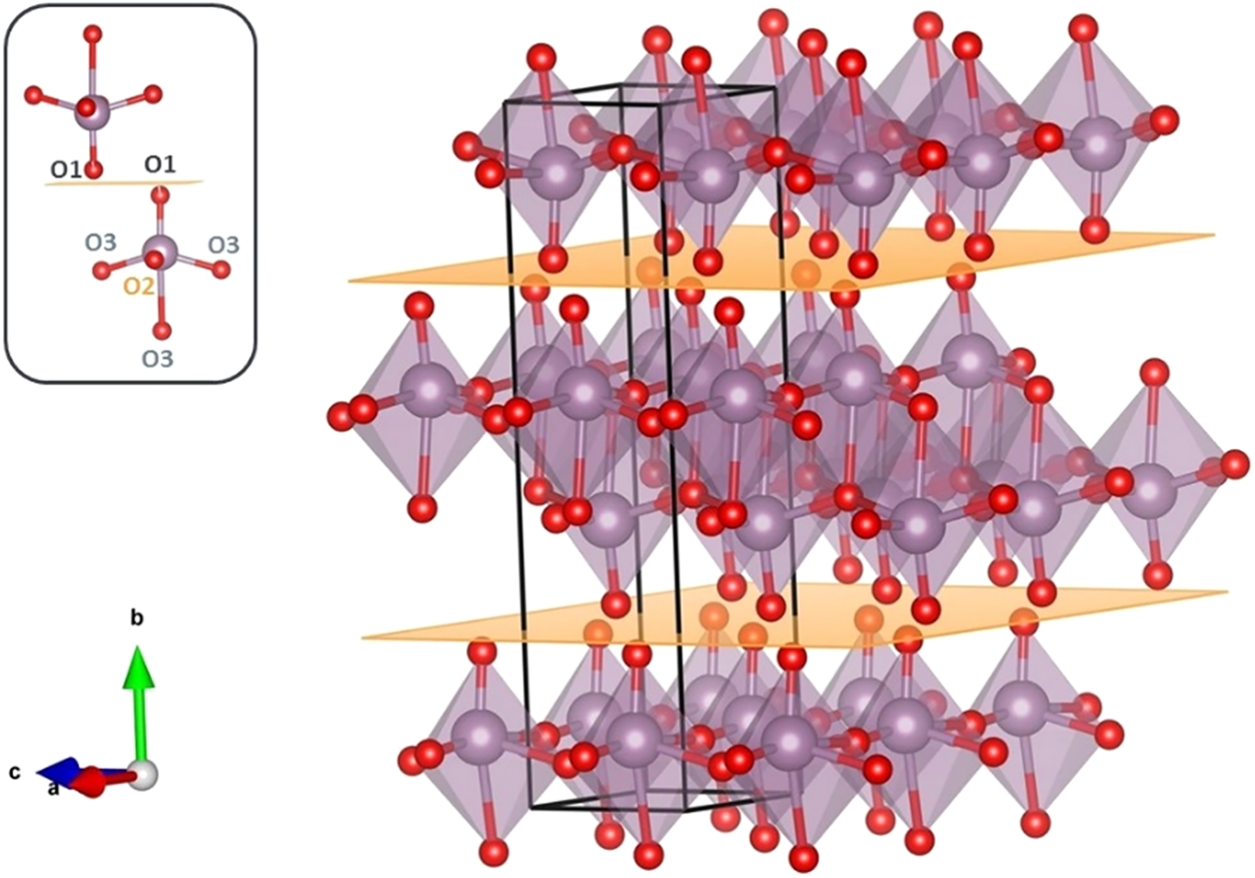
3D visualization of α–MoO_3_ drawn with VESTA software suite.^47^ Unit cell parameters *b* are from Table 1; unit cell parameters *a* and *c* were taken from ref 48, and kept as constraints in the 3D visualization. Red spheres are oxygen atoms, violet spheres Mo atoms. Yellow horizontal planes represent the two van der Waals gaps per α–MoO_3_ unit-cell along the [010] direction. The unit cell is defined by the continuous black line. The inset shows the oxygen sites in the MoO_6_ octahedra.

To explore the trends of hydrogen interaction with MoO_3_, we conducted several density functional theory (DFT) calculations for hydrogen sorption into the bulk of α-MoO_3_ using the solid-state software suite CASTEP,^49^ which employs 3D periodic boundary conditions. The calculations employed an approach that included an on-the-fly generated ultrasoft pseudopotential, the Perdew–Burke–Ernzerhof (PBE) exchange-correlation functional,^50^ Koelling-Harmon relativistic treatment, an energy cutoff of 490 eV, and the Monkhorst–Pack scheme for Brillouin zone sampling with a spacing of 0.04 Å^–1^. To accurately account for the weak interlayer interactions along the [010] direction, as well as the hydrogen bonding introduced by OH groups formed upon hydrogen insertion into the α-MoO_3_ unit cell, the DFT calculations were corrected using the Tkatchenko–Scheffler (TS) dispersion method.^51^ This semiempirical, environment-dependent correction effectively describes van der Waals forces and hydrogen bonding, both of which play a crucial role in determining the structural and energetic stability of oxide and layered material systems.^52–55^ The close agreement between the experimentally determined unit cell parameters of α–MoO_3_ (*a* = 3.963 Å, *b* = 13.855 Å, and *c* = 3.696 Å), and those obtained from our calculations (*a* = 3.929 Å, *b* = 14.165 Å, and *c* = 3.686 Å) supports the validity of the DFT approach adopted in this study.^56^

The systems were fully optimized, including both atomic positions and all six unit cell parameters, with the unit cell symmetry reduced to the *P*1 symmetry group, until the convergence criteria were satisfied. A variable number (*n*_H_ = 1–4) of hydrogen atoms were attached to the oxygen atoms of type O1, with four O1 atoms per unit cell. A maximum of two hydrogen atoms can bond to each O1, with an O–H bond distance of 1.42 Å. When two hydrogen atoms are bonded to the same O1, they maintain a separation of 1.25 Å between them (see Figure 1 for the identification of O1–O3 oxygen atoms). Depending on the configuration and the interactions between the hydrogen atoms, the O1 atom, and neighboring atoms, either an OH group, an H_2_ molecule, or an H_2_O molecule may form. The hydrogen atoms covalently bonded to O1 may also form hydrogen bonds with neighboring oxygen atoms. In the case of OH and H_2_O, the hydrogen atoms can form hydrogen bonds with neighboring oxygen atoms, and these molecules may desorb from the layers. While all possible configurations of the hydrogen atoms were not exhaustively enumerated, a limited but relevant set of hydrogen-MoO_3_ configurations was considered for each specific case of *n*_H_.

The stability of the hydrogen modified α–MoO_3_ unit cell is characterized by the hydrogen sorption energy, which was calculated as

1 $$E_{sorp}=\left(E_{nH+MoO_{3}}^{Tot}-E_{MoO_{3}}^{Tot}-nE_{H}^{Tot}\right)/n$$

where *E*_nH + MoO_3__^Tot^ is the total energy of the MoO_3_ unit cell with *n* hydrogen atoms absorbed, *E*_MoO_3__^Tot^ is the total energy of the MoO_3_ unit cell, and *E*_H_^Tot^ is the energy of a single hydrogen. The total energy of the hydrogen atom is calculated by the CASTEP software using 3D periodic boundary conditions, with the hydrogen atom placed in the center of an empty cubic box with an edge length of 30 Å. The same calculation scheme was used for the MoO_3_-based systems, except that the reciprocal space grid was reduced to the Γ point.

The theoretical hydrogen storage capacity of α–MoO_3_, as a function of the number of hydrogen atoms n_*H*_ incorporated into its unit cell, is expressed in terms of weight percent (wt %) of hydrogen

2 $$wt\;\%\;\left(H\right)=\frac{n_{H}\cdot m_{H}}{Z\cdot m_{MoO_{3}}+n_{H}\cdot m_{H}}\times 100$$

where *m*_H_ = 1.008 g/mol and *m*_MoO_3__ = 143.95 g/mol are the masses of the hydrogen atom and of the MoO_3_ formula unit, respectively. The parameter *Z* (equal to 4 in this case) represents the number of formula units per unit cell of α–MoO_3_.

## 3 Results and Discussion

### 3.1 GIXRD Analysis

A set of samples, including a 34 nm thick α–MoO_3_ film, α–MoO_3_ (34 nm)/Al_*x*_O_*y*_ (13 nm) stack, and α–MoO_3_ (34 nm)/Al_*x*_O_*y*_ (39 nm) stack, were fabricated on silicon substrates using the ALD technique.

GIXRD patterns of the samples are shown in Figure 2. As we previously detailed in ref 37, the fresh MoO_3_ films are mostly composed of the orthorhombic α–MoO_3_ polymorph, with a minor weight fraction of the β–MoO_3_ polymorph. The unit cell parameter *b* of the α–MoO_3_, calculated experimentally using GIXRD, is 13.91 Å in all of the films (Table 1), consistent with that of molybdite.^48^ Hence, the capping layer of Al_*x*_O_*y*_ introduced to reduce exposure of the active layer to air, did not affect its internal structure.

**Figure 2:**
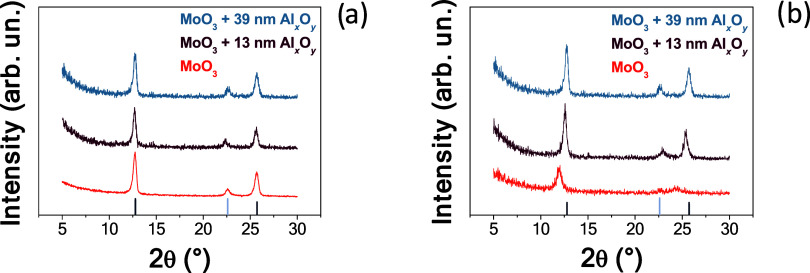
(a) GIXRD patterns of the fresh untreated specimens. (b) GIXRD patterns of the films after 12 min H-plasma treatment. The vertical bars represent the (0*k*0) reflections of α–MoO_3_ (dark blue) and the (011) reflection of the β–MoO_3_ polymorph (light blue).

**Table 1:** Unit Cell Parameter *b* (Å) in the Analyzed Samples, before and after 12 min Hydrogen Plasma Treatment at 200 mTorr, as Derived from GIXRD, and Thickness of the α–MoO_3_ Layer, and the Al_*x*_O_*y*_ Capping Layer before and after the H-Plasma Treatment, as Derived from Spectroscopic Ellipsometry Measurements

	unit cell parameter *b* (Å)		α–MoO_3_ thickness (nm)		Al_*x*_O_y_ thickness (nm)	
sample	before	after	before	after	before	after
MoO_3_	13.91	14.81	34	31		
MoO_3_ + 13 nm Al_*x*_O_*y*_	13.91	14.08	34	34	13	13
MoO_3_ + 39 nm Al_*x*_O_*y*_	13.91	13.91	34	34	39	39

Figure 2b clearly shows that changes happen in the films following the hydrogen plasma treatment of 12 min.

The disappearance of the β–polymorph was observed in the film with no Al_*x*_O_*y*_ layer. Most importantly, a shift toward lower angles of the (0*k*0) reflections (Table 1) occurred in the films with no Al_*x*_O_*y*_ layer, and in that with a nominal 13 nm Al_*x*_O_*y*_ capping layer, with the shift being more pronounced in the former. This latter behavior indicates an expansion in the *b*-axis of the molybdite structure, which is more pronounced in the film with no Al_*x*_O_*y*_ layer, as reported in Table 1 – plasma treatment slightly etches the surface of this latter film too, the thickness decreasing of around 3 nm, from 34 to 31 nm. This expansion in the *b*-axis (qualitatively) reflects a hydrogen absorption process in the molybdite structure. On the other hand, no changes were observed in the film with a nominal 30 nm Al_*x*_O_*y*_ capping layer, as also shown in Figure 2a,b, and Table 1, therefore, no hydrogen absorption was experienced by this specimen under these experimental conditions. Also, the 12 min H-plasma treatment did not affect the thickness of the Al_*x*_O_*y*_ capping layers, as retrieved by SE measurements, listed in Table 1.

The *b*-axis expansion of the hydrogenated samples was monitored over air exposure times. The α–MoO_3_ film without the Al_*x*_O_*y*_ capping layer fully reverted to its original value after 90 days (see Figure S1a,b in the Supporting Information). Such recovery was not observed when the hydrogen-loaded α–MoO_3_ film without the Al_*x*_O_*y*_ capping layer was stored in a controlled nitrogen atmosphere, as demonstrated in Figure S2. These results demonstrate that the Al_*x*_O_*y*_ layer effectively serves as a barrier, preventing any interaction with ambient oxygen.^34^

Given these results, we will exclusively focus on the α–MoO_3_ film with a nominal 13 nm Al_*x*_O_*y*_ capping layer. Indeed, such a recovery in the *b*-axis of the molybdite did not occur with the Al_*x*_O_*y*_ layer, as shown in Figure 3, indicating that the absorbed hydrogen remains stable in the α–MoO_3_ structure.

**Figure 3:**
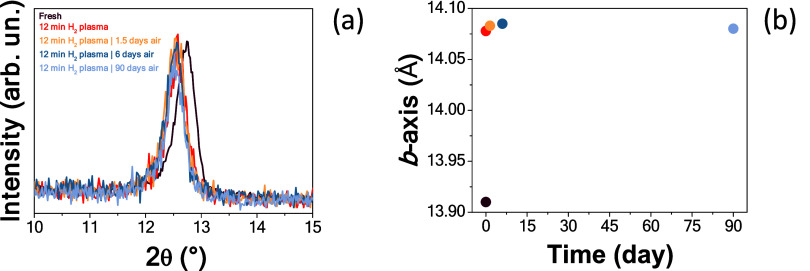
(a) GIXRD patterns [showing only the (020) reflection] of the α–MoO_3_ film with a nominal 13 nm Al_*x*_O_*y*_ capping layer before, after H-plasma treatment, and after being exposed to air for up to 90 days. (b) Corresponding evolution of the α–MoO_3_ *b*-axis over the same specimens as in (a).

The optimized stack α–MoO_3_ (34 nm)/Al_*x*_O_*y*_ (13 nm) was also subjected to H-plasma treatments with different durations. As shown in Figure 4a, an incremental shift in the *b*-axis occurs with increasing treatment time up to 24 min, after which it remains stable up to 96 min. This indicates that the shift in the *b*-axis of α–MoO_3_ is virtually the same as that observed after 24 min of H-plasma treatment. This suggests (qualitatively) that hydrogen absorption reaches a plateau at 24 min H-plasma treatment. It is worth noting that Al_*x*_O_*y*_ remains almost unaffected by H-plasma treatment up to 96 min. This observation is supported by ellipsometry measurements performed after hydrogen plasma treatments of varying durations (12, 24, 48, and 96 min), which consistently yielded an Al_*x*_O_*y*_ layer thickness of approximately 13 nm. Furthermore, SEM micrographs of the Al_*x*_O_*y*_/MoO_3_ stack treated at these time points (Figure S3) revealed changes only in the α–MoO_3_ grain structure, with no significant Al_*x*_O_*y*_ cracking or delamination observed.

**Figure 4:**
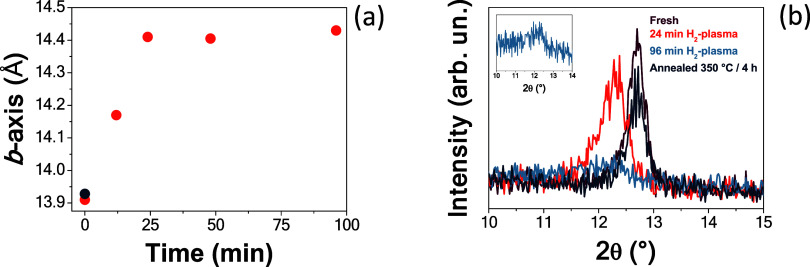
(a) Unit cell parameter *b* of the α–MoO_3_ film with a nominal 13 nm Al_*x*_O_*y*_ capping layer after different H-plasma treatment times (orange circles), and after annealing in a N_2_ atmosphere at 350 °C for 4 h (dark blue circle). (b) GIXRD patterns [showing only the (020) reflection] of the α–MoO_3_ film with a nominal 13 nm Al_*x*_O_*y*_ capping layer after different H-plasma treatment times, and after being annealed under a N_2_ atmosphere at 350 °C/4 h. The inset shows a magnification of the GIXRD pattern of the film treated with H-plasma for 96 min.

Additionally, as shown in Figure S3a–c, the α–MoO_3_ surface morphology remains virtually unchanged after 12 min of H-plasma treatment, and shows only slight changes after 24 min. Conversely, after 96 min of treatment (Figure S3d), some changes become visible: the surface exhibits a more pronounced clustering of grains. This morphological change does not improve the crystallinity of the film, as demonstrated in Figure 4b. Therefore, amorphization appears to be the dominant effect induced by extended hydrogen plasma exposure.

Moreover, as seen in Figure 4b and its inset, the longest H-plasma treatment (96 min) broadens the (020) α-MoO_3_ reflection, suggesting a reduction in the size of the coherently scattering domains, along with a likely partial (hydrogen induced) amorphization.^57^

### 3.2 ERDA Analysis

Quantitative ERDA results are shown in Figure 5. As observed, a small amount of hydrogen (1.3 × 10^16^ H atoms, first column in Figure 5) is present in the α–MoO_3_ film with the Al_*x*_O_*y*_ capping layer. This hydrogen content reflects the total amount measured in the full stack (α–MoO_3_ + Al_*x*_O_*y*_), and may originate from residual species of the metal–organic Mo and Al precursors used during the deposition process. The hydrogen content in the Al_*x*_O_*y*_ capping layer alone is approximately 1.5 × 10^15^ atoms, that in the α–MoO_3_ layer is 1.2 × 10^16^ atoms.

**Figure 5:**
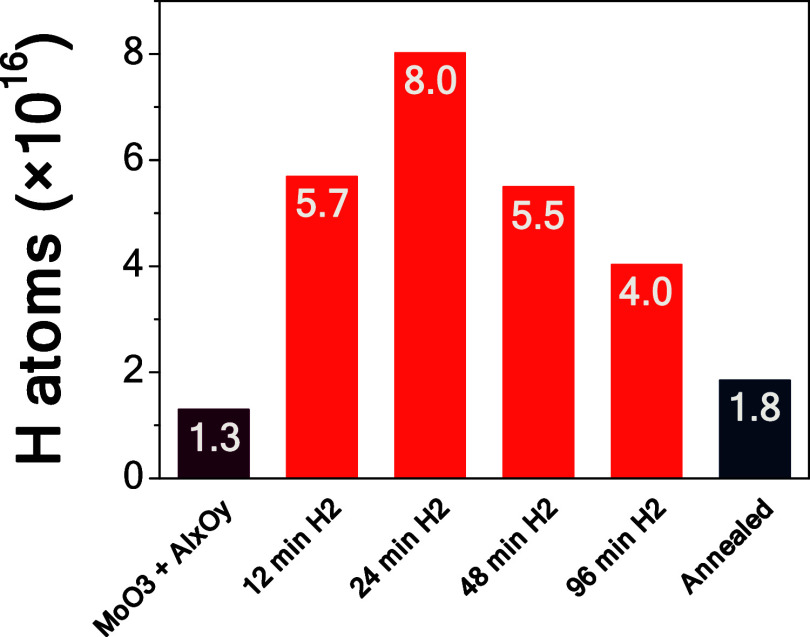
Hydrogen atoms absorbed in 1 cm^2^ of a 34 nm thick α–MoO_3_ film, as determined by ERDA measurements. The columns show the total H atoms measured in the fresh α–MoO_3_ + Al_*x*_O_*y*_ stack (i.e., 34 + 13 nm, respectively) and after different H-plasma treatments. The amount of hydrogen atoms in the sample treated with 24 min H-plasma after release by annealing (at 350 °C/4 h) is also reported in the last column.

When the MoO_3_ films are subjected to the H-plasma treatment, they clearly absorb hydrogen in their bulk, thus corroborating the computational predictions of Goncalves et al., who proposed that such absorption in MoO_3_ is energetically favorable, even at ambient temperature and low H_2_ pressures.^18^ Indeed, consistent with the qualitative GIXRD measurements, there is an increase in the hydrogen absorption up to 24 min of H-plasma treatment, with a maximum of 8.0 × 10^16^ H atoms. Considering that the Al_*x*_O_*y*_ control film treated with 24 min H-plasma contains approximately 1.0 × 10^16^ H atoms, and the fresh MoO_3_/Al_*x*_O_*y*_ stack contains 1.3 × 10^16^ H atoms, we can deduce that the “*real*” amount of H atoms absorbed in the MoO_3_ layer is 5.7 × 10^16^. Given that in 1 cm^2^ of a 34 nm thick α–MoO_3_ film there are 6.4 × 10^16^ O1 sites (and, consequently, 1.3 × 10^17^ O2 sites), this implies that H atoms have at least 6.4 × 10^16^ sites (in that volume of film) in which they can be absorbed. While literature reports – as discussed in more detail in Section 3.4 – that H atoms might also be absorbed at O2 sites,^58^ and that up to two H atoms can be accommodated at an O1 site,^59^ our results with the 24 min H-plasma treatment, coupled with those from GIXRD, indicate that we have closely approached the amount of H atoms that can be absorbed by O1 sites in the α–MoO_3_ film.

Besides, the volumetric hydrogen storage capacity of our 34 nm film treated 24 min H-plasma is 28 kg·m^–3^. While this value is below both the theoretical maximum of fully hydrogenated H_2_MoO_3_ (95 kg·m^–3^), and the typical range reported for metal hydrides (i.e., between 90 and 150 kg·m^–3^, see also Table S2),^60^ it was achieved under mild plasma conditions and without the use of high-pressure cycling, indicating significant promise for further optimization. This value is comparable to that of pressurized steel cylinders (typically 36 kg·m^–3^, but achieved at pressures as high as 700 bar), which are currently the most widely accepted hydrogen storage technology.^61^ Moreover, the adaptable deposition process we used allows for the growth of films on flexible and rollable substrates, which makes this technique viable for various practical applications. Literature reports several nonstoichiometric H_*x*_MoO_3_ phases,^62^ with the stoichiometric phase at *x* = 2.0 having the maximum hydrogen content.^63^ This implies a maximum theoretical volumetric capacity of 95 kg·m^–3^. Given that this capacity is within the range reported for metal hydrides, α–MoO_3_ shows promises as a hydrogen storage material.

When the films are treated with more than 24 min of H-plasma, the hydrogen absorption starts to decrease. The film treated for 48 min with H-plasma absorbed 5.5 × 10^16^ H atoms, and that treated for 96 min absorbed 4.0 × 10^16^ H atoms. Although the GIXRD measurements reported a similar expansion in the *b*-axis for 24, 48, and 96 min of treatment, the number of H atoms loaded in the specimens decreased with H-plasma treatment of 48 and 96 min. A likely explanation is that prolonged exposure to H-plasma is detrimental to the film’s microstructure. As observed with GIXRD, the α–MoO_3_ (020) reflection broadened with the H-plasma treatment time, its breadth increased, and its intensity decreased. While relying on only a family of reflections is an approximation, this broadening indicates a drastic increase in the (micro)­structural defects, along with a decrease in the size of the coherently scattering domains,^64^ which may be accompanied by the partial, hydrogen induced, amorphization of the α–MoO_3_ film.^57^ This lack of structural order, in turn, may reduce the number of available sites for hydrogen absorption within the film.

Finally, annealing the film treated with H-plasma for 24 min at 350 °C for 4 h under a nitrogen atmosphere proved effective for total hydrogen desorption – consistent with the dehydrogenation temperature of MgH_2_ (>300 °C).^65,66^ While hydrogen plasma treatment represents an unconventional method for hydrogen storage, it is important to note that conventional hydrogen absorption processes typically require high temperatures (≥300 °C) and elevated pressures (≥7 bar),^36^ making them inherently energy-intensive.

The desorption process is further confirmed by the plot of the evolution of optical properties during the absorption/desorption process, as displayed in Figure S4. The refractive index *n* nearly fully recovers after annealing at 350 °C/4 h the film treated for 24 min with H-plasma.

### 3.3 Reversibility of Hydrogenation

To examine the reversibility of the hydrogenation process, the specimen treated with H-plasma for 24 min at 200 mTorr was annealed at 350 °C for 4 h under a nitrogen atmosphere (Figure 6). After annealing, the specimen was subjected to another 24 min hydrogen plasma treatment. GIXRD measurements were recorded to calculate the percentage expansion, and the following recovering, of the unit cell parameter *b*. As shown in Figure 6, the unit cell parameter *b* expands, and totally recovers itself to the starting value after repeated cycles of 24 min hydrogen plasma treatment and annealing at 350 °C for 4 h under a nitrogen atmosphere. This also confirms that, with a 24 min H-plasma treatment, the layered orthorhombic structure of the parent α–MoO_3_ is retained, thus making the process reversible. This is in contrast to the irreversible changes observed during the initial lithiation of a pristine α–MoO_3_ electrode.^67^

**Figure 6:**
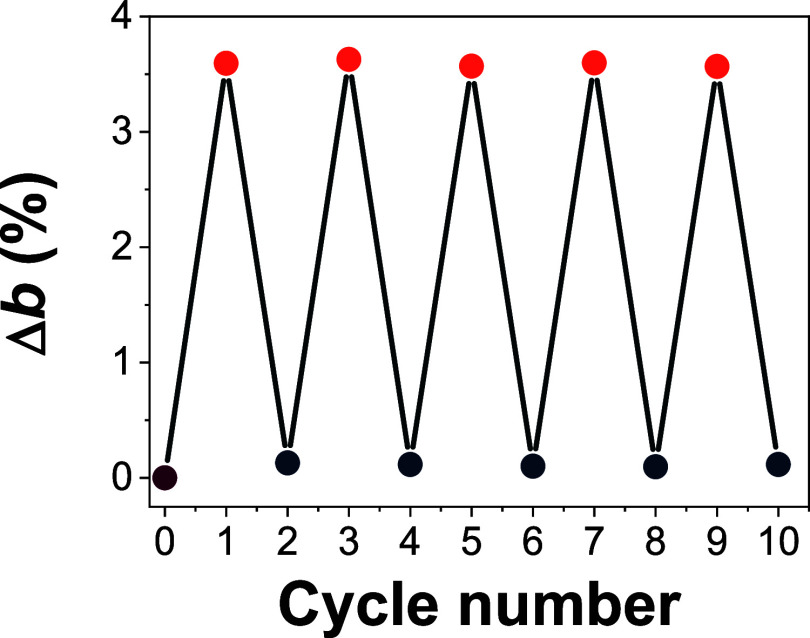
Percentage in the expansion of the α–MoO_3_ unit cell parameter *b*, depicting the H_2_ storage/recovery switches with repeated 24 min plasma at 200 mTorr cycles (orange circles)/350 °C for 4 h under a N_2_ atmosphere (dark blue circles) cycles. The purple circle represents the untreated specimen.

### 3.4 Proposed Absorption Mechanism

While there is plenty of literature, the hydrogen surface coverage of α-MoO_3_ still remains a topic of debate. Some authors, aided by density functional theory (DFT) calculations on hydrogen absorption at the α–MoO_3_(010) surface, state that hydrogen atoms absorb most favorably at the O1 oxygen on both perfect and O1-defective surfaces. The next most favorable site is the O2 oxygen, while the least favorable adsorption site is the O3 oxygen.^68^ Other studies, using DFT, predict the O2 oxygen to be the most advantageous site for hydrogen absorption, followed by the terminal O1 oxygen, with the O3 oxygen being the least preferred.^69^ More recently, Lei and Chen, using the DFT + U method, revealed that a single hydrogen atom absorbs itself preferentially at the O2 oxygen site, while the terminal O1 oxygen becomes the preferred position for accommodating two hydrogen atoms. In other words, hydrogen atoms preferentially occupy the asymmetrical O2 oxygen at low coverage, whereas at high coverage, they favor the terminal O1 site.^59^

However, all these cited studies refer to hydrogen sorption on the surface of the metal oxide. On the contrary, hydrogen in our system diffuses into the bulk region of the material, rather than interacting exclusively at the surface. Only a few studies have investigated hydrogen absorption in the bulk region of α–MoO_3_.^18,70^

Indeed, according to Pez et al., the terminal O1 oxygen exhibits stronger electron affinity due to its unsaturated valence. Additionally, hydrogen atom coordination in the bulk shows significant differences compared to the (010) surface. In the bulk, the terminal oxygen is the most favorable site for hydrogen absorption. Furthermore, hydrogen atoms in the bulk interact with the two terminal O1 oxygen atoms in adjacent layers via weaker hydrogen bonds, which does not occur on the (010) surface.^70^ The same authors also suggested a preferential diffusion pathway for hydrogen atoms absorbed in bulk α–MoO_3_. Initially, the hydrogen atom is absorbed onto a terminal O1 oxygen. It then transfers to another terminal O1 oxygen site in the adjacent layer through the van der Waals gap. Subsequently, it migrates within the layer to an adjacent asymmetric O2 site, before moving to the next neighboring asymmetric site. The diffusion process concludes at another terminal O1 oxygen site.^70^ They state that O1 and O2 sites, interacting with H atoms with nearly equal bond strength, may both be populated upon H atom absorption. This likely explains why the precise locations of H atoms in the α–MoO_3_ structure vary across different experiments, depending on specimen preparation.^70^ These findings are supported by a recent study by Goncalves and coauthors, who, based on DFT studies, identified bulk MoO_3_ as a highly promising candidate material for reversible hydrogen storage. They found that hydrogen absorption is energetically favorable even at ambient temperature and low H_2_ pressures.^18^

Based on these findings, and knowing that the distance between two equivalent terminal O1–O1 oxygen sites – vertically aligned along the [010] – in our α–MoO_3_ is 7.16 Å before the H-plasma treatment, and 7.40 Å after 24 min of hydrogen plasma treatment at 200 mTorr, we observe an expansion in the van der Waals gap of around 0.24 Å. Thus, knowing that the hydrogen atoms become protonic when coordinated to oxygen, besides molybdenum reduction, it is highly predictable that hydrogen is absorbed as H^+^ at O1 oxygen sites, as suggested by Pez et al.^70^ By analogy with their work, and considering our experimental data (*i.e*. an expansion in the [0*k*0] α–MoO_3_ crystallographic direction upon H-plasma treatment), we propose that hydrogen is preferentially absorbed on the terminal O1 oxygen atoms. While hydrogen diffusion to the O2 asymmetric sites, as proposed in [58,70], cannot be excluded *a priori*, we lack experimental data on both the [*h*00] crystallographic direction and structural data (i.e., Mo–O distances) upon H-plasma treatment to validate this. Moreover, the reversibility of the process (*vide supra*) suggests that no significant structural changes occurred, implying that hydrogen absorption most likely occurs in the van der Waals gap, potentially at O1 sites.^46,71^

To evaluate the behavior of the MoO_3_ host in the presence of sorbed hydrogen atoms, DFT calculations were performed for various degrees of hydrogen sorption by inserting *n*_H_ = 0–4 hydrogen atoms per unit cell, corresponding to a composition of H_*x*_MoO_3_, with *x* = 0–1. The results show that, when a single hydrogen atom is inserted, it preferentially forms a covalent bond with the O1-type oxygen atoms. The analysis of hydrogen sorption energy (Figure 7) indicates that all constructed systems are stable, with sorption energies *E*_sorp_ < 0 (see Table 2). This stability is attributed to the formation of new O1–H covalent bonds, and the presence of 1 to 3 hydrogen bonds (*n*_HB_ = 1–3, Table 2) between the inserted hydrogen atoms and nearby oxygen atoms. Attempts to insert hydrogen at O2– or O3–-type oxygen sites result in structural destabilization, as indicated by the full purple diamond symbol in Figure 7. Consequently, our study focuses exclusively on hydrogen absorption at O1 sites. Hirshfeld charge analysis supports the covalent nature of the O1–H bonds through charge transfer from hydrogen to O1 and nearby atoms. For example, upon insertion of two hydrogen atoms (*n*_h_ = 2), the net charges on adjacent Mo and O1 atoms change from 0.76 |e| to 0.72 |e|, and from −0.21 |e| to −0.25 |e|, respectively. Each hydrogen atom donates approximately 0.88 |e|, primarily to the O1 atoms and neighboring Mo atoms, confirming substantial electron sharing and the covalent character of the O1–H interaction.

**Figure 7:**
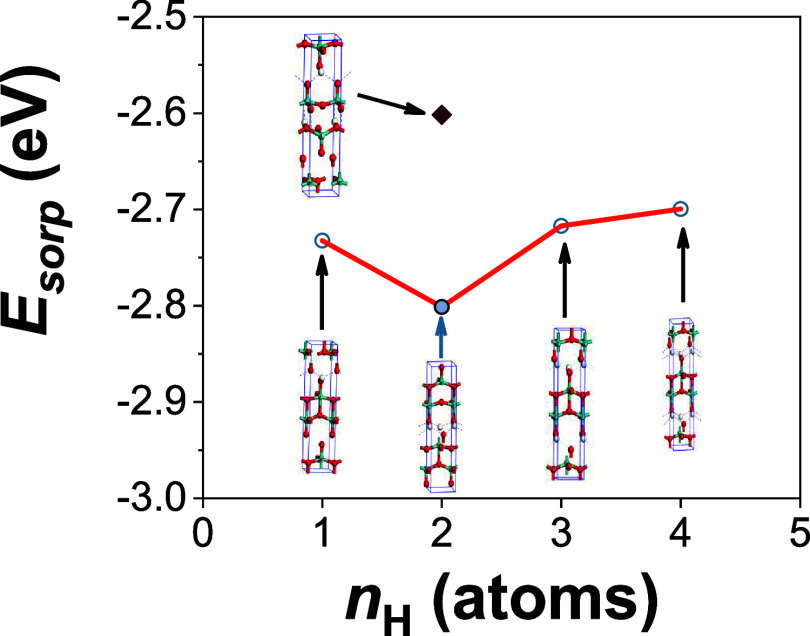
Hydrogen sorption energy (*E*_sorp_) as a function of the number of inserted hydrogen atoms (*n*_H_) per unit cell of α-MoO_3_, along with corresponding representative ball-and-stick atomic configurations. In the ball-and-stick models, cyan, red, and white spheres represent molybdenum, oxygen, and hydrogen atoms, respectively. Dashed blue lines indicate hydrogen bonds. The orange continuous line is a guide-to-the-eye, and links the convex hull (*Q*_hull_) points corresponding to the most stable configurations. The light-blue-filled circle marks the minimum energy point on the Q_hull_ line. A case where two hydrogen atoms are absorbed onto O1 and O2 oxygen sites is indicated by full purple diamond symbol.

**Table 2:** Hydrogen Sorption Energy of the Most Stable Polymorphs (*Q*_hull_), Symmetry Group, and Unit Cell Parameters for *n*_H_ Hydrogen Atoms Inserted into a α–MoO_3_ Unit Cell

					unit cell parameters		
number of inserted hydrogen atoms per unit cell (*n*_H_)	theoretical hydrogen storage capacity (wt %)^a^	Number of hydrogen bonds per hydrogen atom (*n*_HB_)	*Q*_hull_ of hydrogen sorption energy, *E*_sorp_ (eV)	symmetry group	*a* (Å)	*b* (Å)	*c* (Å)
0	0.000	0		*Pbmn*	3.929	14.265	3.686
1	0.175	2	–2.732	*Pm*	3.994	14.931	3.748
2	0.350	2	–2.801	*Pmn*2_1_	4.114	15.458	3.784
3	0.522	2	–2.717	*Pm*	3.791	15.948	3.731
4	0.695	2	–2.699	*Pnma*	3.786	16.069	3.750

a Values calculated using eq 2.

A minimum in the hydrogen sorption energy is present along the *Q*_hull_ curve (the curve connecting the lowest energy points for each *n*_H_), see Figure 7. This configuration occurs at *n*_H_ = 2 (*E*_sorp_= – 2.801 eV), and corresponds to two hydrogen atoms positioned on that bilayer formed by two MoO_6_ octahedra – both facing a shared interbilayer space. Adding another hydrogen atom to the O1 site from the adjacent interlayer space destabilizes the system, as the hydrogen sorption energy increases to 2.717 eV. Further destabilization (*E*_sorp_ = −2.699 eV) occurs when the interlayer space is completed by a hydrogen atom that forms a bond with the last unsaturated O1 site in the unit cell. For *n*_H_ ≤ 4, each hydrogen atom forms two bifurcated hydrogen bonds with two neighboring oxygen atoms.

Finally, as listed in Table 2, the unit cell parameter *b* increases as hydrogen atoms are inserted, from 14.265 Å in unmodified α–MoO_3_ (*i.e., n*_H_ = 0), to 16.069 Å for *n*_H_ = 4 (DFT-calculated). Thus, the expansion predicted by the DFT simulations agrees (qualitatively) with the GIXRD experimental results, where we attributed the observed expansion to hydrogen absorption on O1 sites.

## 4 Conclusions

Hydrogen has the potential to play a significant role in the transition to a more sustainable and decarbonized energy system. However, its actual storage systems are of high cost, low energy density, and undergo safety concerns. While materials-based storage methods display interesting alternatives, a strong candidate for storing hydrogen still lacks. In this work, we have proposed [010] oriented α–MoO_3_ thin films as a potential alternative material for solid-state hydrogen storage. It has been shown that hydrogen plasma is a suitable way to hydrogenate, at room temperature and at relatively low pressure, α–MoO_3_ thin films. By means of XRD and DFT analysis, we have proposed that hydrogen located itself in the van der Waals gaps along the [010] crystallographic direction. ERDA analysis supported the GIXRD findings, providing a quantitative measure of the hydrogen atoms absorbed in the α–MoO_3_ thin film. The results highlighted an increase in hydrogen absorption with up to 24 min of H-plasma treatment, followed by a decrease for longer treatment durations, aligning with the structural changes observed through GIXRD. The film treated for 24 min with H-plasma achieved a volumetric hydrogen storage capacity of 28 kg·m^–3^. While this value is lower than the theoretical maximum for fully hydrogenated H_2_MoO_3_ (95 kg·m^–3^), it is totally comparable to that of pressurized steel cylinders (i.e., typically 36 kg·m^–3^, but attained at high pressure up to 700 bar). This shows its viability for practical use and highlights significant potential for further optimization. The hydrogen sorption process has been found to be totally reversible upon annealing under a nitrogen atmosphere at 350 °C/4 h, and recoverable after repeated cycles. Consistent with GIXRD observations, DFT calculations demonstrated that hydrogen bonds preferentially to O1 sites in α–MoO_3_, resulting in an expansion along the *b*-axis. The flexibility of the deposition technique allows for the growth of films on a variety of substrates, including flexible and rollable ones, enhancing the material’s potential for integration into diverse technological contexts. While we do not assert that our system is presently suitable for large-scale implementation, we consider that our research offers significant insights into the hydrogen storage capabilities of α–MoO_3_, potentially laying the groundwork for subsequent material optimization and technological progress in this filed.

## Supplementary Material



## Notes

Additional discussion on hydrogen desorption in α–MoO_3_ films without Al_*x*_O_*y*_ capping, including: GIXRD patterns before and after hydrogen plasma treatment and ambient storage, and: evolution of the *b*-axis during desorption. SEM micrographs of the α–MoO_3_/Al_*x*_O_*y*_ stack following hydrogen plasma treatments of 0, 12, 24, and 96 min. Additional discussion on hydrogen absorption in the stack α–MoO_3_/Al_*x*_O_*y*_, including: refractive index evolution of the α–MoO_3_/Al_*x*_O_*y*_ stack before and after hydrogen absorption and desorption, measured by spectroscopic ellipsometry. Comparative data on volumetric and gravimetric hydrogen storage capacities of benchmark materials (PDF)

## #

Vittorianna Tasco is currently seconded at the European Research Council Executive Agency of the European Commission. Her views expressed in this paper are purely those of the writer and may not in any circumstance be regarded as stating an official position of the European Commission
