# Supplementary material for: Solid-State Hydrogen Storage in Atomic Layer Deposited α‑MoO3 Thin Films
Source: Energy Fuels. 2025 Jun 4;39(23):11388–97. doi: 10.1021/acs.energyfuels.5c01159 (PMC12172136; doi:10.1021/acs.energyfuels.5c01159)
Supplement: Supplementary file 1 [file ef5c01159_si_001.pdf]

Supporting Information to:

Solid-state hydrogen storage in atomic layer deposited α−MoO_3 thin films

David Maria Tobaldi,[a,*] Salvatore Mirabella,[b] Gianluca Balestra,[a,c] Daniela Lorenzo,[a] Vittorianna Tasco,[‡,a] Maria Grazia Manera,[d] Adriana Passaseo,[a] Marco Esposito,[a] Andreea Neacsu,[e] Viorel Chihaia,[e] and Massimo Cuscunà[a,*]

[a]*CNR Nanotec, Institute of Nanotechnology, University Campus Ecotekne, Via per Monteroni, 73100 Lecce, Italy*

[b]*Dipartimento di Fisica e Astronomia "Ettore Majorana", Università di Catania, Cittadella Universitaria, Via Santa Sofia 64, 95123 Catania, Italy*

[c]*Department of Mathematics and Physics ''Ennio De Giorgi", University of Salento, c/o Campus Ecotekne, Via Monteroni, 73100, Lecce, Italy*

[d]*CNR IMM, Institute for Microelectronic and Microsystems, University Campus Ecotekne, Via per Monteroni, 73100 Lecce, Italy*

[e]*Institute of Physical Chemistry "Ilie Murgulescu", Romanian Academy, Splaiul Independentei 202, 060021 Bucharest, Romania*

*Corresponding authors.

E-mail addresses: david.tobaldi@nanotec.cnr.it; massimo.cuscuna@nanotec.cnr.it.

‡ Vittorianna Tasco is currently seconded at the European Research Council Executive Agency of the European Commission. Her views expressed in this paper are purely those of the writer and may not in any circumstance be regarded as stating an official position of the European Commission.

S1. Hydrogen absorption in the α−MoO_3 film without Al_xO_y capping layer

Figure S1a shows the GIXRD patterns of the α−MoO_3 film, without an Al_xO_y capping layer, before and after a 12-minute hydrogen-plasma treatment at 200 mTorr, and the subsequent shift of the (020) reflection to higher angles after being stored in air for up to 90 days. Figure S1b reports the evolution of the *b*-axis of the α−MoO_3 displayed in Figure S1a. As observed, there is virtually a complete recovery of the initial *b*-axis value, qualitatively implying a total desorption of the absorbed hydrogen. We suggest that absorbed hydrogen might react with ambient oxygen, leading to the formation of desorbed water,[S1] thus restoring the original length of α−MoO_3 unit cell parameter *b*. To test this hypothesis, the α−MoO_3 film was exposed to 24-hour N_2 flux immediately after the H-plasma treatment (Figure S2). As shown in Figure S2, there was virtually no shift in the (020) reflections: this allows us to reasonably hypothesise that, after the N_2 flux, hydrogen remained stored in the α−MoO_3 layered structure.

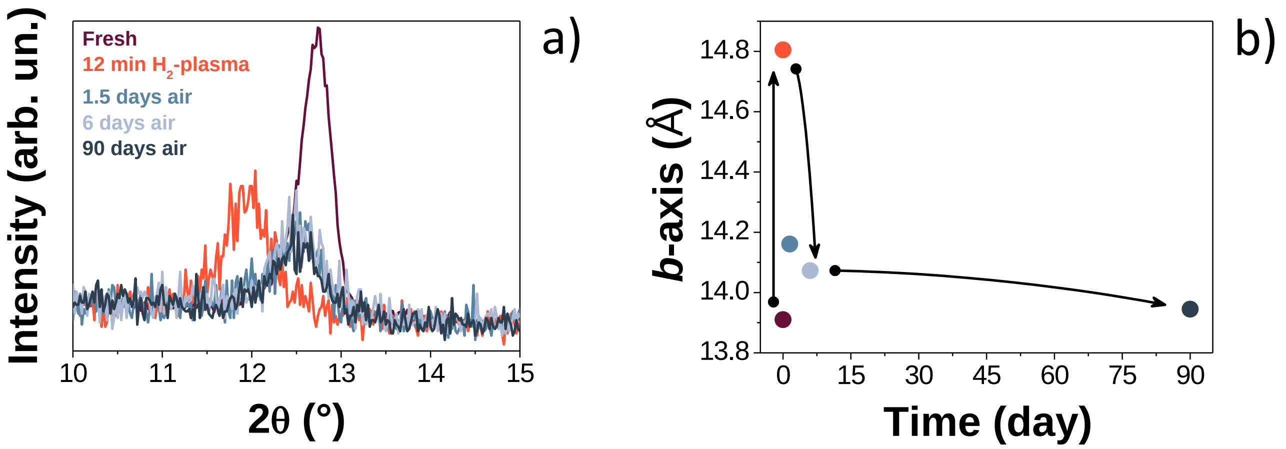

Figure S1 – a) GIXRD patterns [showing only the (020) reflection] of the fresh α−MoO_3 film with no Al_xO_y capping layer, the same film after a 12 minutes H-plasma treatment, and after being stored in air up to 90 days. b) Evolution of the unit cell parameter *b* of the fresh α−MoO_3 film (purple circle), after being treated for 12 minutes with H-plasma (orange circle), and after being stored in air up to 90 days. The arrows are a guide-for-the-eye.

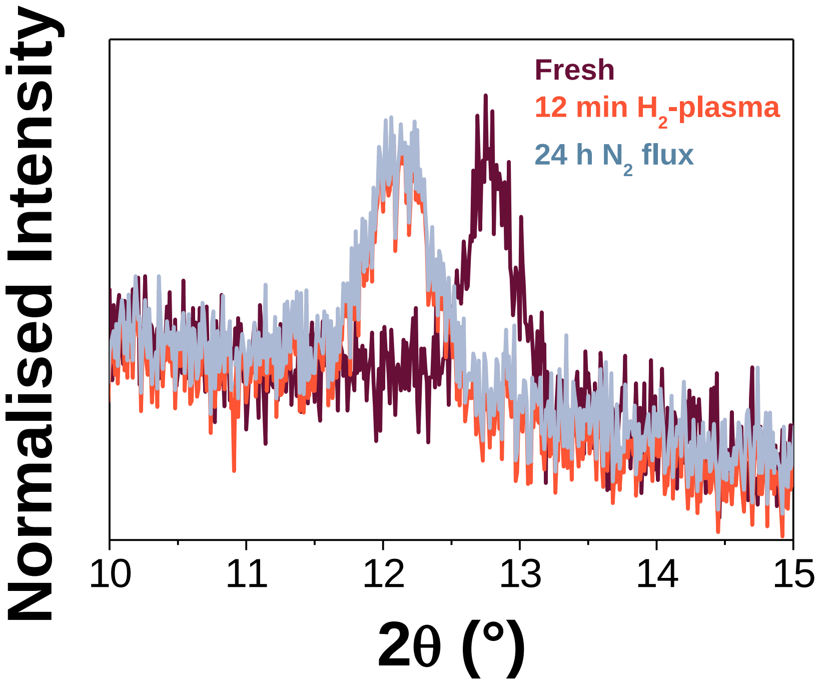

Figure S2 – GIXRD patterns [showing only the (020) reflection] of the fresh α−MoO_3 film with no Al_xO_y capping layer, the same film after a 12 minutes H-plasma treatment, and after being subjected to 24 hours N_2 flux.

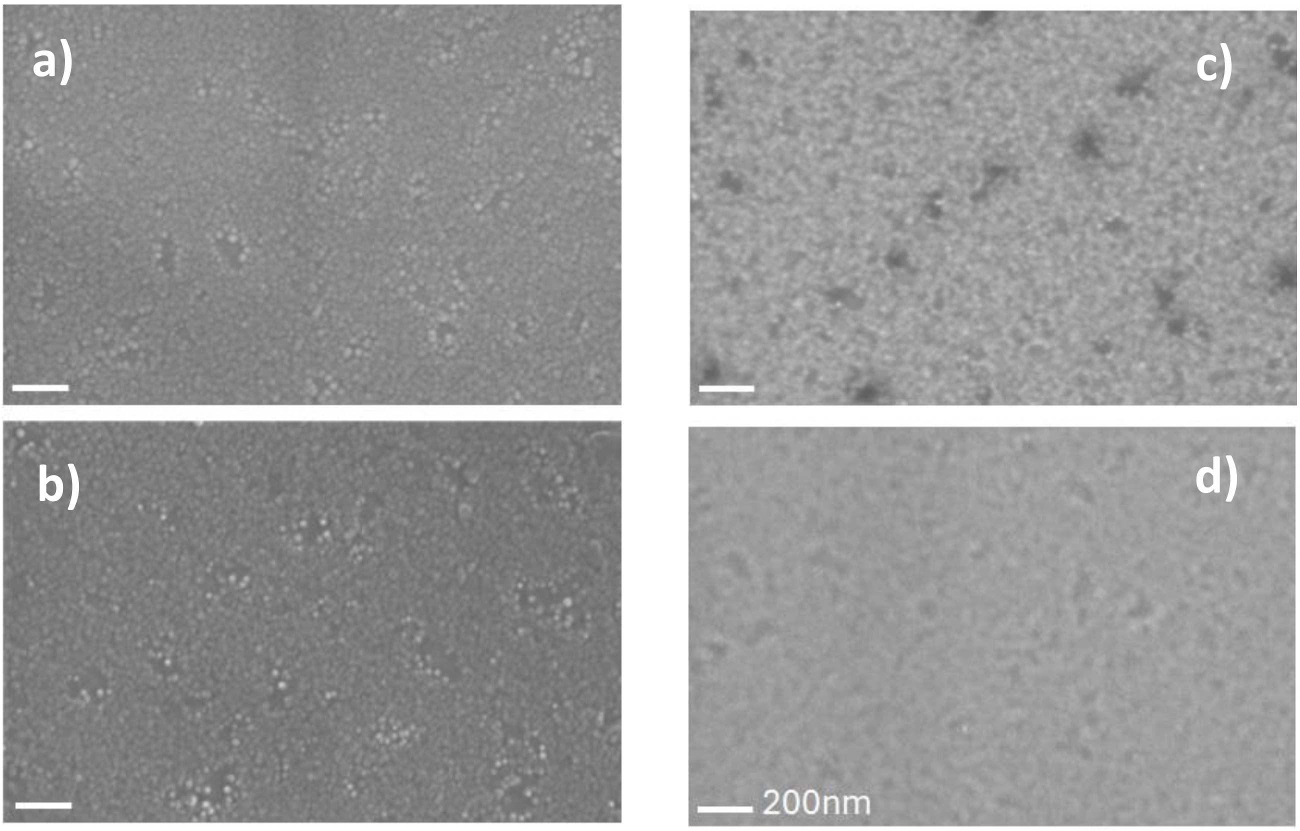

Figure S3 – SEM micrographs showing the surface of: (a) untreated Al_xO_y/MoO_3 stack; (b) Al_xO_y/MoO_3 stack after 12 minutes of H-plasma treatment; (c) Al_xO_y/MoO_3 stack after 24 minutes of H-plasma treatment; (d) Al_xO_y/MoO_3 stack after 96 minutes of H-plasma treatment. The scale bar is the same in all the micrographs, representing 200 nm.

S2. Hydrogen absorption in the stack α−MoO_3/Al_xO_y

Optical analysis was assessed by spectroscopic ellipsometry to determine the refractive index of the α−MoO_3 layer within the stack α−MoO_3/Al_xO_y, both before and after hydrogen absorption and desorption. As shown in Figure S3, there is a noticeable decrease in the refractive index across the visible to near-infrared (Vis/NIR) range following hydrogen absorption. This observation is in good agreement with the expansion of the α−MoO_3 unit cell parameter *b*, as proven by GIXRD. In a simplified picture, the expansion of the film leads to a reduction in its density, which in turn lowers its refractive index. After hydrogen desorption through annealing at 350 °C / 4 h, the refractive index nearly reverts to its original value. A similar behaviour was observed by GIXRD measurements presented in the main text.

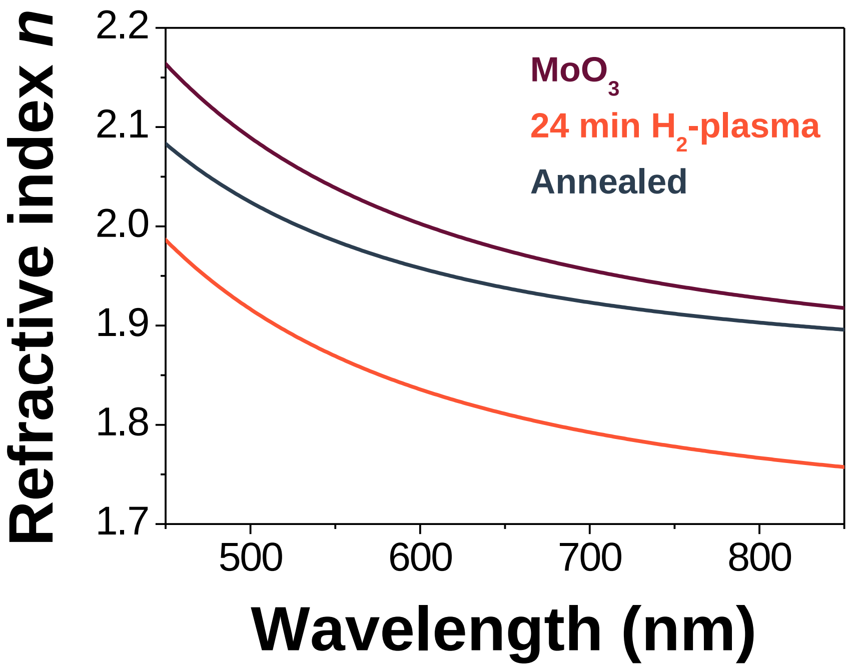

Figure S4 – Evolution of the MoO_3 refractive index *n* in the stack MoO_3/Al_xO_y before, after 24minutes H-plasma treatment, and after the annealing process.

Table S1 – Comparison of volumetric hydrogen storage capacity in different materials.

Material	Hydrogen storage capacity (Volumetric or Gravimetric)	Notes	Reference
MgH_2	105 $kg_{H2}.m^{-3}$	Sorption at 5 bar, 620 K; Desorption at > 573 K	S2, S3
$NaAlH_4$	38 $kg_{H2}.m^{-3}$	Calculated	S4
Mg_2FeH_6	150 $kg_{H2}.m^{-3}$	Theoretical, difficult to synthesise	S5
AlH_3	50 $kg_{H2}.m^{-3}$	Desorption at T > 473 K	S6
Boron nitride	20 $kg_{H2}.m^{-3}$	Sorption at 5 MPa, 298 K	S7
Activated carbon monoliths	39.3 $kg_{H2}.m^{-3}$	Sorption at 4 MPa, 77 K	S8
Activated carbon monoliths	41 $kg_{H2}.m^{-3}$	Sorption at 77 K	S9
MOF	40 $kg_{H2}.m^{-3}$	Sorption at 77 K	S10
MOF-5	71 $kg_{H2}.m^{-3}$	Sorption at 11 MPa, 77 K	S11
Zeolites	30 $kg_{H2}.m^{-3}$	Sorption at 15 bar, 77 K	S12
Ti-grafted graphene oxide	64 $kg_{H2}.m^{-3}$	Computational study	S13
MoS_2 bulk	1.2 wt%	Gravimetric storage capacity	S14
MoS_2 exfoliated	2.4 wt%	Gravimetric storage capacity	S14
Porous MoS_2	6.43–7.52 wt%	Gravimetric storage capacity; Computational study. Porosity influences the wt% values	S15
**MoO_3 thin film**	**28**	**Sorption at 200 mTorr, room temperature; Desorption at 623 K**	**This work**

Supporting Information References

(S1) Ou, J. Z.; Campbell, J. L.; Yao, D.; Wlodarski, W.; Kalantar-zadeh, K. In Situ Raman Spectroscopy of H_2 Gas Interaction with Layered MoO_3. *J. Phys. Chem. C* **2011**, *115* (21), 10757–10763. https://doi.org/10.1021/jp202123a.

(S2) Züttel, A. Materials for Hydrogen Storage. *Mater. Today* **2003**, *6* (9), 24–33. https://doi.org/10.1016/S1369-7021(03)00922-2.

(S3) Wan, H.; Fang, D.; Zhou, S.; Yang, X.; Dai, Y.; Ran, L.; Chen, Y.; Pan, F. Enhanced Dehydrogenation Properties and Mechanism Analysis of MgH2 Solid Solution Containing Fe Nano-Catalyst. *Int. J. Hydrog. Energy* **2023**, *48* (87), 34180–34191. https://doi.org/10.1016/j.ijhydene.2023.05.201.

(S4) Lozano, G. A.; Ranong, C. N.; Bellosta Von Colbe, J. M.; Bormann, R.; Hapke, J.; Fieg, G.; Klassen, T.; Dornheim, M. Optimization of Hydrogen Storage Tubular Tanks Based on Light Weight Hydrides. *Int. J. Hydrog. Energy* **2012**, *37* (3), 2825–2834. https://doi.org/10.1016/j.ijhydene.2011.03.043.

(S5) Polanski, M.; Płociński, T.; Kunce, I.; Bystrzycki, J. Dynamic Synthesis of Ternary Mg2FeH6. *Int. J. Hydrog. Energy* **2010**, *35* (3), 1257–1266. https://doi.org/10.1016/j.ijhydene.2009.09.010.

(S6) Ahluwalia, R. K.; Hua, T. Q.; Peng, J. K. Automotive Storage of Hydrogen in Alane. *Int. J. Hydrog. Energy* **2009**, *34* (18), 7731–7740. https://doi.org/10.1016/j.ijhydene.2009.07.013.

(S7) Tokarev, A.; Kjeang, E.; Cannon, M.; Bessarabov, D. Theoretical Limit of Reversible Hydrogen Storage Capacity for Pristine and Oxygen-Doped Boron Nitride. *Int. J. Hydrog. Energy* **2016**, *41* (38), 16984–16991. https://doi.org/10.1016/j.ijhydene.2016.07.010.

(S8) Jordá-Beneyto, M.; Lozano-Castelló, D.; Suárez-García, F.; Cazorla-Amorós, D.; Linares-Solano, Á. Advanced Activated Carbon Monoliths and Activated Carbons for Hydrogen Storage. *Microporous Mesoporous Mater.* **2008**, *112* (1–3), 235–242. https://doi.org/10.1016/j.micromeso.2007.09.034.

(S9) Xiao, J.; Hu, M.; Cossement, D.; Bénard, P.; Chahine, R. Finite Element Simulation for Charge–Discharge Cycle of Cryo-Adsorptive Hydrogen Storage on Activated Carbon. *Int. J. Hydrog. Energy* **2012**, *37* (17), 12947–12959. https://doi.org/10.1016/j.ijhydene.2012.05.072.

(S10) Broom, D. P.; Webb, C. J.; Fanourgakis, G. S.; Froudakis, G. E.; Trikalitis, P. N.; Hirscher, M. Concepts for Improving Hydrogen Storage in Nanoporous Materials. *Int. J. Hydrog. Energy* **2019**, *44* (15), 7768–7779. https://doi.org/10.1016/j.ijhydene.2019.01.224.

(S11) Yang, J.; Wang, H.; Dong, X.; Gong, M. Performances Comparison of Adsorption Hydrogen Storage Tanks at a Wide Temperature and Pressure Zone. *Int. J. Hydrog. Energy* **2023**, *48* (69), 26881–26893. https://doi.org/10.1016/j.ijhydene.2023.03.351.

(S12) Langmi, H. W.; Book, D.; Walton, A.; Johnson, S. R.; Al-Mamouri, M. M.; Speight, J. D.; Edwards, P. P.; Harris, I. R.; Anderson, P. A. Hydrogen Storage in Ion-Exchanged Zeolites. *J. Alloys Compd.* **2005**, *404–406*, 637–642. https://doi.org/10.1016/j.jallcom.2004.12.193.

(S13) Wang, L.; Lee, K.; Sun, Y.-Y.; Lucking, M.; Chen, Z.; Zhao, J. J.; Zhang, S. B. Graphene Oxide as an Ideal Substrate for Hydrogen Storage. *ACS Nano* **2009**, *3* (10), 2995–3000. https://doi.org/10.1021/nn900667s.

(S14) Altuntepe, A.; Erkan, S.; Olğar, M. A.; Çelik, S.; Zan, R. Hydrogen Storage Capacity of Two-Dimensional MoS2. *Int. J. Hydrog. Energy* **2024**, *56*, 690–698. https://doi.org/10.1016/j.ijhydene.2023.12.120.

(S15) de Oliveira, F. B.; da Rosa, A. L. Dichalcogenides and Difulfides Nanostructures for Hydrogen Storage. arXiv 2024. https://doi.org/10.48550/ARXIV.2404.16761.
